# Synergistic Rescue of Nonsense Mutant Tumor Suppressor p53 by Combination Treatment with Aminoglycosides and Mdm2 Inhibitors

**DOI:** 10.3389/fonc.2017.00323

**Published:** 2018-01-04

**Authors:** Meiqiongzi Zhang, Angelos Heldin, Mireia Palomar-Siles, Susanne Öhlin, Vladimir J. N. Bykov, Klas G. Wiman

**Affiliations:** ^1^Department of Oncology-Pathology, Cancer Center Karolinska (CCK), Karolinska Institutet, Stockholm, Sweden

**Keywords:** p53, nonsense mutation, aminoglycosides, MDM2 inhibitors, readthrough

## Abstract

The tumor suppressor gene *TP53* is inactivated by mutation in a large fraction of human tumors. Around 10% of *TP53* mutations are nonsense mutations that lead to premature termination of translation and expression of truncated unstable and non-functional p53 protein. Aminoglycosides G418 (geneticin) and gentamicin have been shown to induce translational readthrough and expression of full-length p53. However, aminoglycosides have severe side effects that limit their clinical use. Here, we show that combination treatment with a proteasome inhibitor or compounds that disrupt p53-Mdm2 binding can synergistically enhance levels of full-length p53 upon aminoglycoside-induced readthrough of R213X nonsense mutant p53. Full-length p53 expressed upon combination treatment is functionally active as assessed by upregulation of p53 target genes, suppression of cell growth, and induction of cell death. Thus, our results demonstrate that combination treatment with aminoglycosides and compounds that inhibit p53 degradation is synergistic and can provide significantly improved efficacy of readthrough when compared with aminoglycosides alone. This may have implications for future cancer therapy based on reactivation of nonsense mutant *TP53*.

## Introduction

The tumor suppressor p53 (TP53) is a transcription factor that regulates a wide range of cellular processes including cell cycle progression, DNA repair, metabolism, apoptosis, and senescence (1, 2). Under normal conditions, p53 levels are low due to proteasomal degradation upon ubiquitination by the E3 ligase Mdm2, encoded by the p53 target gene *MDM2* (3). In response to various forms of cellular stress, p53 accumulates and transactivates target genes such as *CDKN1A* (*P21*), *GADD45, BAX, PUMA, NOXA*, and *TIGAR*, leading to a p53-dependent biological response. One crucial function of p53 as tumor suppressor is the elimination of incipient tumor cells upon oncogenic stress (4).

The *TP53* gene is mutated in a large fraction of human tumors (5, 6). The majority of *TP53* mutations (74%) are missense mutations that result in single amino acid substitutions in p53 (7, 8). These mutations are clustered in the DNA-binding p53 core domain. *TP53* mutation in several types of tumors is associated with poor therapy response and survival (9, 10). Therefore, p53 is an important clinical prognostic marker.

Around 10% of *TP53* mutations are nonsense mutations (6–8) that give rise to premature termination codons (PTCs), resulting in the expression of unstable truncated p53 or complete lack of p53 expression due to nonsense-mediated mRNA decay (NMD) (11). R213X and R196X are the two most frequent nonsense *TP53* mutations in human tumors, and R213X is present in about 1% of all human tumors (7, 8), corresponding to roughly 141,000 new cancer cases worldwide 2012 and estimated 236,000 cases in 2030 (12, 13). R213X is the 6th most common *TP53* mutation in 12 common cancer types and the 2nd most common *TP53* mutation in lung squamous cell carcinoma after R158L (6).

Recent studies have also indicated that p53 is a promising therapeutic target. Our group has previously discovered the small molecules PRIMA-1 and APR-246 (PRIMA-1^MET^) that restore wild-type p53 confirmation and function to missense mutant p53 protein, and trigger tumor cell death by apoptosis (14, 15). APR-246 is currently being tested in a phase II proof-of-concept study in high-grade serous ovarian cancer. A number of other missense mutant p53-targeting compounds have been identified through various strategies (14).

Aminoglycoside antibiotics inhibit bacterial protein synthesis through targeting the 16S rRNA of the bacterial ribosome, resulting in mismatch of tRNA anti-codons with both sense and stop codons and impairment of normal translation (16–18). Gentamicin is used in the clinic for a wide range of bacterial infections (19), while G418 applications are restricted to laboratory research to select genetically modified cells. Aminoglycosides were first found to suppress premature stop codons in yeast (20). More recently, aminoglycoside antibiotics G418 and gentamicin have been shown to promote translational readthrough of PTCs and restore expression of full-length proteins in mammalian cells. This has been demonstrated for the cystic fibrosis gene (21, 22), the *DMD* gene (23), the *ATM* gene (24, 25), and the *APC* gene in colon cancer (26). The molecular mechanism of the translational readthrough is not fully understood. Importantly, normal termination codons are not significantly affected by aminoglycosides in mammalian cells, even though they are extensively affected in bacteria (17, 27). This is consistent with data showing that the mechanisms for normal termination and premature termination are different (28). G418 and gentamicin were also shown to induce full-length p53 protein in HDQ-P1 breast carcinoma cells homozygous for the R213X nonsense mutant *TP53* allele, leading to upregulation of p53 target genes *CDKN1A* (*P21*) and *BAX* at the mRNA level (29). Similarly, an aminoglycoside derivative was shown to induce readthrough of *TP53* nonsense mutations Q192X, R213X, and E298X, resulting in expression of full-length p53 with biological activity as assessed by induction of p53 target genes *CDKN1A* (*P21*) and *BAX* and R213X-dependent cell death (30).

The clinical use of aminoglycosides is limited by their nephrotoxicity and ototoxicity (31). Therefore, it is highly desirable from clinical point of view to identify novel potent inducers of translational readthrough with more favorable toxicity profiles, or develop combination treatment that allows lower and non-toxic doses of aminoglycosides. Here, we have studied translational readthrough of nonsense mutant *TP53* by aminoglycosides further and show that the proteasome inhibitor bortezomib as well as the p53-Mdm2 inhibitors nutlin-3a and MI-773 can enhance the levels of full-length p53 and potentiate tumor cell death upon treatment with aminoglycosides.

## Materials and Methods

### Cells and Cell Culture

HDQ-P1 human breast cancer cells carry a homozygous nonsense mutation at codon 213 (CGA to TGA; R213X) in the *TP53* gene (32). HDQ-P1 cells (DSMZ, Braunschweig, Germany) were cultured in DMEM low-glucose medium (Hyclone, Logan, UT, USA) supplemented with 10% fetal bovine serum (Hyclone, Logan, UT, USA) and 2.5 µg/ml plasmocin (Invivogen, Toulouse, France). H1299 lung adenocarcinoma cells (ATCC, Wesel, Germany) are p53 null (33). They were grown in RPMI-1640 medium (Hyclone, Logan, UT, USA) supplemented with 10% fetal bovine serum and 2.5 µg/ml plasmocin. For stably transfected H1299 cells, 1 µg/ml puromycin was added in the culture medium. HCT116 wtp53^+/+^ colon cancer cells (34) were cultured in McCoy medium (Hyclone, Logan, UT, USA) supplemented with 10% fetal bovine serum and 2.55 µg/ml plasmocin.

### Plasmid Constructs

The plasmid pCMV-puro-bam with the CMV promoter and the puromycin resistance gene were used as backbone and empty vector. The plasmid pCMV-puro-bam p53 R213X was created by inserting the p53 coding sequence with a nonsense mutation at codon 213 downstream of the CMV promoter in pCMV-puro-bam. The plasmid pCMV-puro-bam R213XΔ*C*-EGFP was generated by inserting the first 213 codons of R213X p53 fused in frame with the EGFP coding sequence downstream of the CMV promoter. All plasmid constructs were made by GenScript (Genscript, Piscataway, NJ, USA).

### Transient Transfection and Selection of Transfectants

H1299 cells were plated in six-well plates at a density of 200,000 cells per well. Next day cells were transfected with 1 µg DNA constructs per well by using Lipofectamine 2000 (Invitrogen, Carlsbad, CA, USA) according to the manufacturer’s protocol. For selection of stably transfected cells, cells were transfected as indicated above and cultured in the presence of 1 µg/ml puromycin.

### Western Blotting

Cells were plated in six-well plates at a density of 300,000 cells per well (HDQ-P1) or 150,000 cells per well (H1299 and HCT116 wtp53^+/+^). Cells were allowed to adhere overnight and were then treated with 100, 200, and 400 µM of G418 (HyClone Laboratories, Logan, UT, USA) or with 2 mM of gentamicin (Gibco/Life Technologies, Stockholm, Sweden) for 72 h. For combination treatment with either G418 or gentamicin, different concentrations of bortezomib, nutlin-3a, or MI-773 were added for 48 h (bortezomib) or 72 h (nutlin-3a and MI-773). Bortezomib, nutlin-3a, and MI-773 were from Sigma-Aldrich (Stockholm, Sweden). Cells were harvested and lysed with RIPA buffer, 20–50 µg of protein were loaded on 10% SDS/PAGE gels (Life Technologies, Stockholm, Sweden) and run in 1× MOPS (Life Technologies, Stockholm, Sweden). Gels were blotted on nitrocellulose membranes using iBlot (Life Technologies, Stockholm, Sweden). Membranes were blocked in PBS-T with 5% milk and then incubated with antibodies against p53 (DO-1, Santa Cruz, CA, USA; PAb421, Calbiochem Merk, Darmstadt, Germany; HR231, a kind gift from Dr. Thierry Soussi, Department of Oncology-Pathology, Karolinska Institutet, Stockholm, Sweden), p21 (p21 F-5, Santa Cruz, CA, USA; p21 Waf1/Cip1, Cell Signaling Technology, Leiden, Netherlands), or Wig-1 (FJ1, raised against a C-terminal Wig-1 peptide, F. Jerhammar, unpublished). GAPDH (Santa Cruz, CA, USA) and β-actin (Sigma-Aldrich, Stockholm, Sweden) were used as loading controls. Proteins were visualized using HRP-conjugated secondary antibodies and SuperSignal West Femto Maximum Sensitivity Substrate (Thermo Scientific, Stockholm, Sweden) with a CCD camera (Fujifilm, Stockholm, Sweden), analyzed by a Luminescent Image Analyzer LAS-1000 plus, and quantified with Image Quant software (Fujifilm, Stockholm, Sweden).

### RNA Extraction, cDNA Synthesis, and qRT-PCR

RNA was extracted with RNeasy mini kit (Qiagen GmbH, Hilden, Germany) according to the manufacturer’s protocol and quantified by a nanodrop-1000 apparatus (Thermo Scientific, Stockholm, Sweden). cDNA was synthesized using the SuperScript II Reverse Transcriptase (Invitrogen, Carlsbad, CA, USA) according to the manufacturer’s protocol. Real-time PCR (RT-PCR) was carried out in an Applied Biosystems 7500 RT-PCR System sequence detection system version 1.2 (Applied Biosystems, Foster City, CA, USA) according to the manufacturer’s recommendations. TaqMan Gene Expression Assays (TP53: Hs00153340_m1, MDM2: Hs01066930_m1, BAX: Hs00414514_m1, CDKN1A: Hs00355782_m1, BBC3: Hs00248075_m1, c1/c2: Hs01028907_m1, ACTB: Hs99999903_m1, ZMAT3: Hs00536976_m1, PMAIP1: Hs00560402_m1, FAS: Hs00531110_m1, and GAPDH: Hs99999905_m1) (Applied Biosystems, Foster City, CA, USA). Relative gene expression was calculated by the 2 − ΔΔ*Ct* method after normalization to GAPDH levels.

### Immunofluorescence Staining

HDQ-P1, H1299-R213X, and empty vector-transfected H1299 cells were seeded in six-well plates with glass slides, at a density of 300,000 cells per well for HDQ-P1 cells and 200,000 cells per well for H1299 cells. The next day, cells were treated with 100 µM G418. After 72 h treatment, cells were washed in PBS, fixed in 4% paraformaldehyde, permeabilized with 0.2% Triton, and washed in PBS. Anti-p53 antibody PAb421 (Merck, Darmstadt, Germany) was diluted 100 times in 2% BSA and anti-p53 antibody FL393 (Santa Cruz, CA, USA) was diluted 400 times in 2% BSA. Both antibodies were mixed and incubated with the cells for 2 h at room temperature. After washing with PBS, cells were incubated with secondary antibodies conjugated with Alexa Fluor dye (Invitrogen/Thermo Fisher Scientific, Sweden), for 1 h at room temperature, washed with PBS and mounted with HardSet mounting medium with DAPI (Vector Laboratories, CA, USA). Images were obtained with a Zeiss Axioplan 2 microscope with an AxioCam HRm Camera.

### WST-1 Assay

Stably transfected H1299 cells were plated in 96-well plates at a density of 1,500 cells per well. The next day cells were treated with G418 and nutlin-3a at different concentrations. After 72 h, cells were incubated with cell proliferation reagent WST-1 (Roche Diagnostics GmbH, Mannheim, Germany) for 30 min. Absorbance at 450 nm was then determined in a Tecan reader Infinite M1000 (Tecan Austria, Grödig, Austria).

### Flow Cytometry

Nonsense mutant TP53 R213X or empty vector-transfected H1299 cells were seeded in six-well plates at a density of 100,000 cells per well in 2 ml medium. The next day cells were treated with G418 at 200 µM and/or nutlin-3a at 10 µM and incubated for 72 h (all treatment conditions were in duplicates). All cells were collected, washed with PBS, and fixed with ice-cold 100% ethanol overnight at 4°C. The cells were pelleted and the supernatant was discarded. The samples were incubated at 37°C for 30 min in darkness with propidium iodide (0.025 mg/ml) (Sigma-Aldrich, Stockholm, Sweden) and RNase A (0.125 mg/ml) (Sigma-Aldrich, Stockholm, Sweden). 50 µl PBS was added to each sample and the samples were then analyzed with NovoCyte^®^ flow cytometer (ACEA Biosciences, San Diego, CA, USA). Cells were gated by combining forward scatter-height/side scatter-height and forward scatter-height/forward scatter-area gates.

### Statistical Analysis

For comparison of dependent variables, normal distribution of the differences between the compared variables was first assessed. If the distribution was normal, ANOVA model for repeated measurements was employed and for multiple comparisons Tukey or Unequal N HSD *post hoc* tests were applied. If the distribution was not normal, the non-parametric Wilcoxon test was applied. For comparison of independent variables, the Kruskal–Wallis test was applied since the data did not comply with the equal variance requirement for ANOVA model. Statistica 13.0 (Dell Inc., Tulsa, OK, USA) was used for the statistical analysis.

## Results

### Aminoglycosides Induce Readthrough of R213X Nonsense Mutant TP53 and Expression of Full-Length p53

**Figure 1:**
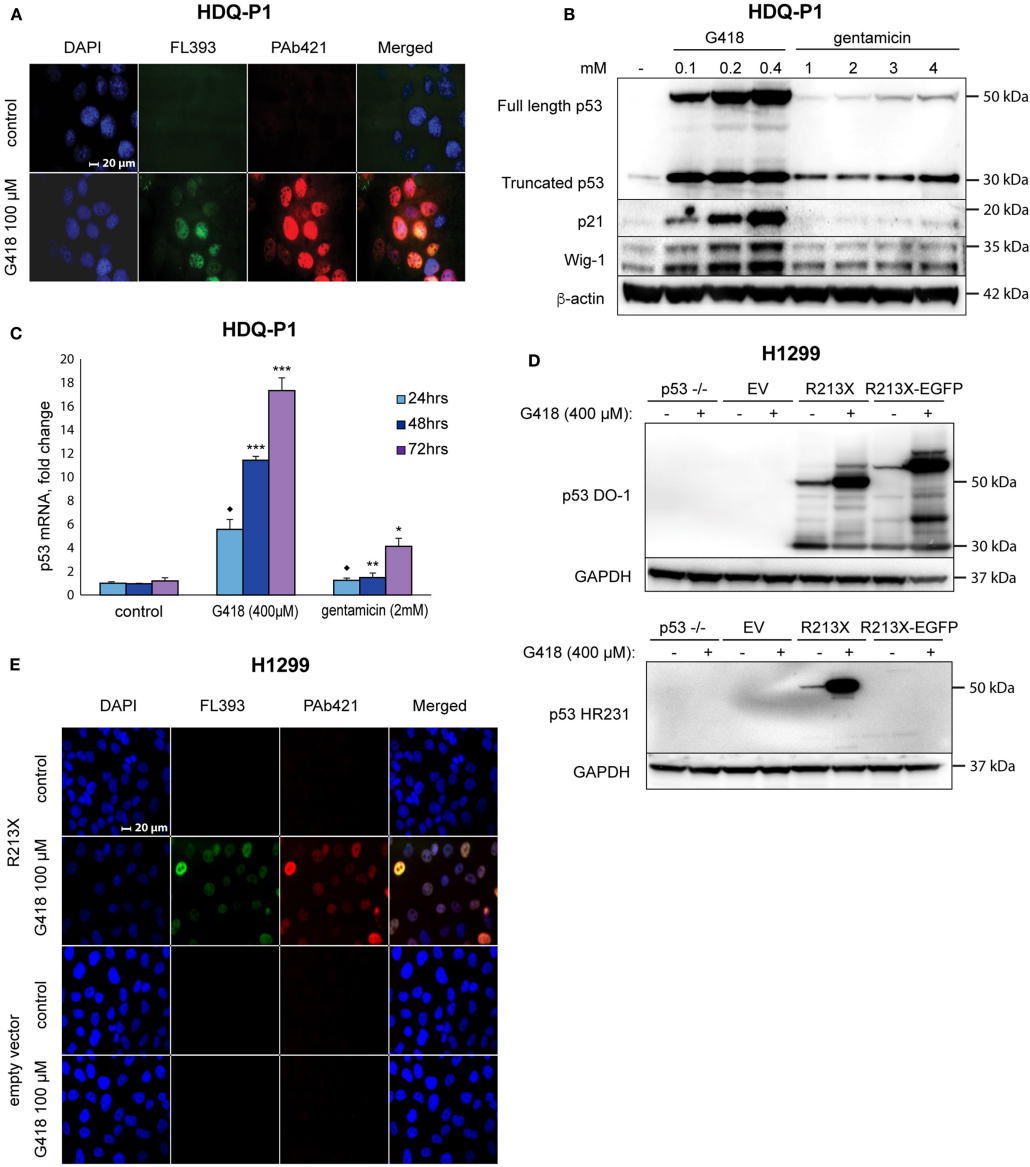
Induction of translational readthrough of R213X nonsense mutant *TP53* in HDQ-P1 and H1299 cells. **(A)** Induction of full-length p53 in HDQ-P1 cells according to immunofluorescence staining with antibodies against full-length p53 (FL393) and the p53 C-terminus (PAb421). **(B)** Induction of full-length p53 and its targets p21 and Wig-1 in HDQ-P1 cells after treatment with G418 and gentamicin as shown by Western blotting. The blot was cut at 25 kDa, the upper part was first probed with p53 antibody and then washed and blotted with antibodies against Wig-1 or β-actin. The lower part was blotted with p21 antibody. **(C)** Significant increase in p53 mRNA level in HDQ-P1 cells after treatment with aminoglycosides compared with control cells at a given time point according to real-time PCR. Three independent experiments were performed in duplicates. Statistical significance was shown using either the parametric Tukey *post hoc* test in the ANOVA model (* = *p* < 0.05, ** = *p* < 0.01, *** = *p* < 0.001) or the non-parametric Wilcoxon matched pairs test (◆ = *p* < 0.05) for samples not fitting normal distribution. **(D)** Induction of full-length p53 in H1299 cells transiently transfected with the R213X or R213XΔ*C*-EGFP constructs or empty vector as shown by Western blotting. Blots were first probed with p53 antibody (DO-1 or HR231) and then stripped and blotted with an antibody against GAPDH. **(E)** Immunofluorescence staining of p53 in H1299 stably transfected with R213X or empty vector upon treatment with G418. Antibodies were FL393 and PAb421 as indicated.

We treated human HDQ-P1 breast carcinoma cells carrying endogenous R213X nonsense mutant *TP53* with G418 and examined expression of full-length p53 protein by immunofluorescence staining with FL393 polyclonal p53 antibody and the monoclonal antibody PAb421 that recognizes an epitope in the p53 C-terminus. Treatment with 400 µM G418 strongly induced p53 according to immunostaining with both the FL393 and PAb421 antibodies (Figure 1A), indicating induction of full-length p53. Western blotting confirmed induction of full-length p53 by G418 in a dose-dependent manner (Figure 1B). Gentamicin also induced full-length p53, although much less efficiently (Figure 1B). These data confirm a previous study showing that aminoglycosides G418 and gentamicin induce translational readthrough of premature stop codon R213X and induce production of full-length p53 protein expression in HDQ-P1 cells (29). Induction of full-length p53 by G418 was accompanied by upregulation of p53 targets p21 (encoded by the *CDKN1A* gene) and Wig-1 (*ZMAT3*) in a concentration-dependent manner, indicating that the recovered full-length p53 protein is at least partially functional as transcription factor (Figure 1B). Gentamicin did not induce significant levels of p21 and Wig-1.

Next, we examined whether G418 and gentamicin affect p53 mRNA levels. Premature stop codons trigger NMD *via* remaining exon-junction complexes (11). Our RT-PCR analysis showed that both G418 and gentamicin induced p53 mRNA levels in a time-dependent manner. G418 treatment resulted in an 18-fold induction of p53 mRNA levels while gentamicin caused a more modest 4-fold induction after 3 days treatment. The differences between all G418 and gentamicin-treated samples and the control samples were statistically significant (*p* < 0.05). For 48 and 72 h treatments, the *post hoc* Tukey test in the ANOVA model was used for statistical analysis. The data for 24 h treatment did not fit normal distribution, and they were therefore analyzed with the Wilcoxon matched pairs test (Figure 1C). The observed increase in p53 mRNA levels is expected since translational readthrough would remove exon-junction complexes and thus inhibit NMD (35). However, we do not exclude the possibility that p53 mRNA level can be enhanced due to increased transcription.

To test this effect in other cells, we transiently transfected human H1299 lung adenocarcinoma cells with either a p53 R213X construct, or a plasmid containing the first 213 codons of R213X p53 fused in frame with EGFP, or empty vector. Western blot analysis with the DO-1 antibody showed expression of full-length p53 in R213X-transfected cells upon G418 treatment (Figure 1D, upper panel). Similarly, we detected G418-mediated induction of the p53 R213XΔ*C*-EGFP fusion protein in H1299 cells transfected with the p53 R213XΔ*C*-EGFP construct (Figure 1D, upper panel). The apparent molecular weight of the p53 R213XΔ*C*-EGFP fusion protein is larger than that of full-length p53 due to the addition of EGFP. The expression of full-length p53 in this setting was confirmed by the p53 C-terminal antibody HR231 (Figure 1D, lower panel). We also generated stable lines of H1299 cells carrying the R213X construct. G418 treatment induced full-length p53 in the H1299-R213X cells as assessed by immunostaining with the C-terminal antibody PAb421 (Figure 1E).

### Induction of Full-Length p53 Is Followed by Upregulation of p53 Target Genes

**Figure 2:**
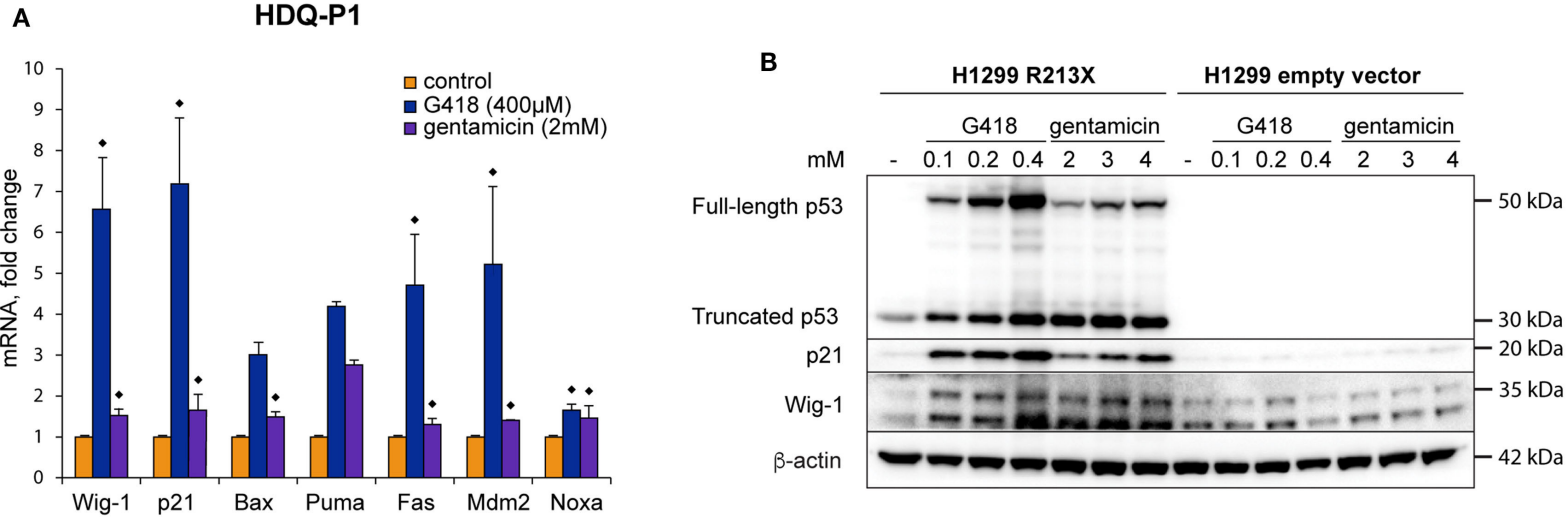
Full-length p53 induced by aminoglycosides is transcriptionally active. **(A)** Induction of p53 target genes at the mRNA level in HDQ-P1 cells after treatment with G418 or gentamicin as shown by real-time PCR. Three independent experiments were carried out with three to six observations for each gene and treatment. Differences in mRNA levels between treatments and control cells were analyzed using the non-parametric Wilcoxon matched pairs test (◆ = *p* < 0.05). **(B)** Western blot analysis showing induction of full-length p53 and p21 in stably transfected H1299 cells upon treatment with G418 or gentamicin. The Western blot was cut at around 25 kDa, the upper part was first probed with p53 antibody and then washed, cut, and blotted with antibodies against β-actin and Wig-1. The lower part was blotted with p21 antibody.

In order to further study if the full-length p53 protein induced by aminoglycosides in HDQ-P1 cells is transcriptionally active, we examined expression of p53 target genes by both RT-PCR and Western blotting. G418 induced p21, Bax, Puma, Fas, Mdm2, Noxa, and Wig-1 at the mRNA level, while gentamicin was less efficient. The differences between G418-treated and control samples were statistically significant for Wig-1, p21, Fas, Mdm2, and Noxa, (*p* < 0.05) but not for Bax and Puma. For gentamicin-treated samples, the differences were statistically significant (*p* < 0.05) for all tested targets except Puma. The Wilcoxon matched pairs test was applied here because the distributions in several groups differed significantly from the normal distribution (Figure 2A). Interestingly, mRNA induction for the studied genes correlated poorly between G418 and gentamicin (*r* = −0.32), suggesting not only differences in the potency between these two antibiotics but possibly also in the activity of the induced full-length p53 protein (see Discussion). We observed the most prominent upregulation of p21 and Wig-1 mRNA by G418, whereas Puma mRNA showed the strongest response after treatment with gentamicin. Western blotting demonstrated that both p21 and Wig-1 were strongly induced by G418, whereas upregulation after gentamicin treatment was relatively weak (Figures 1B and 2B). p21 induction by G418 and gentamicin is likely to be p53-dependent, since the induction is only observed in H1299-R213X cells and not in H1299 cells transfected with empty vector (Figure 2B). These results indicate that aminoglycosides induce full-length p53 that is at least partially functional as transcription factor in HDQ-P1 and H1299-R213X cells.

### Combination Treatment with a Proteasome Inhibitor or p53-Mdm2 Inhibitors Enhances Full-Length p53 Levels and p53 Target Gene Expression

**Figure 3:**
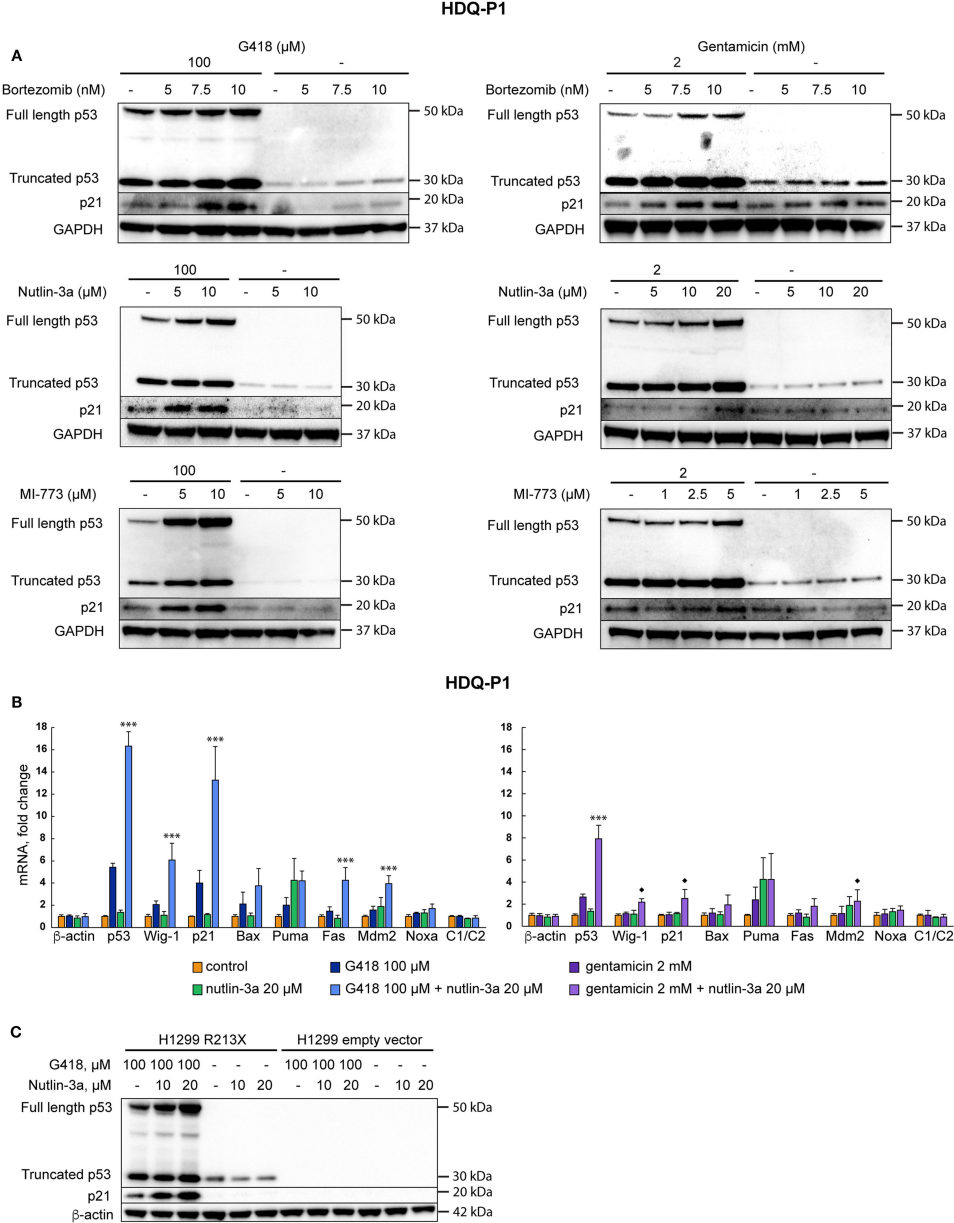
Inhibition of p53 degradation enhances levels of full-length p53 and p53 target genes. **(A)** Induction of readthrough of nonsense mutant *TP53* in HDQ-P1 cells by the combination of aminoglycosides with the proteasome inhibitor bortezomib (upper panel), combination of aminoglycosides with nutlin-3a (middle panel), and the combination of aminoglycosides with MI-773 (lower panel). Left side panels: G418; right side panels: gentamicin. **(B)** Real-time PCR analysis showing upregulation of p53 and p53 target genes at the mRNA level in HDQ-P1 cells. β-actin and c1/c2 were used as negative controls. Left panel: G418; right panel: gentamicin. Three independent experiments were performed in duplicates. Differences between combination treatment and G418 or gentamicin alone for each target were analyzed using either the parametric Tukey *post hoc* test in the ANOVA model (*** = *p* < 0.001) or the non-parametric Wilcoxon matched pairs test (◆ = *p* < 0.05) for samples not fitting normal distribution. **(C)** Combination treatment with G418 and nutlin-3a resulted in synergistic induction of p53 and p21 in H1299 R213X cells as shown by Western blotting. No p53 was detected in empty vector control cells. The blot was cut at around 25 kDa, the upper part was first probed with p53 antibody and then washed and blotted with antibodies against β-actin. The lower part was blotted with p21 antibody.

Next we asked if levels of recovered full-length p53 could be further enhanced by combination treatment with agents that inhibit p53 proteasomal degradation. Combination of G418 with the proteasome inhibitor bortezomib at 10 nM increased full-length p53 levels upon treatment with G418 or gentamicin according to Western blotting (Figure 3A, upper panel). Bortezomib itself did not enhance full-length p53 expression in these cells, but caused a detectable increase in the levels of truncated p53. Combination of aminoglycosides and bortezomib also enhanced expression of the p53 target p21 as shown by Western blotting (Figure 3A, upper panel). We reasoned that specific inhibition of Mdm2-mediated proteasomal degradation of p53 might further enhance p53 levels. Thus, we treated the cells with a combination of aminoglycosides and the Mdm2 inhibitor nutlin-3a (36). This combination showed strong synergy and increased the levels of full-length p53 as well as p21 in a dose-dependent manner (Figure 3A, middle panel). Nutlin-3a itself did not have any effect on the expression of p53 and p21. We also tested MI-773, another inhibitor of p53-Mdm2 binding (37). MI-773 was at least as potent as nutlin-3a in enhancing full-length p53 protein levels in combination with G418 or gentamicin (Figure 3A, lower panel).

We subsequently examined expression of p53 and p53 target genes at the mRNA level upon combination treatment with aminoglycosides and nutlin-3a. As shown in Figure 3B, the combination treatment resulted in a pronounced synergistic increase in expression of p53 as well as p53 targets p21, Fas, Mdm2, and Wig-1 at the mRNA level. Statistically significant differences (*p* < 0.001) between combination treatment and G418 alone were observed for p53, p21, Fas, Mdm2, and Wig-1 according to the *post hoc* Tukey test in the ANOVA model. Combination treatment with gentamicin and nutlin-3a caused a statistically significant increase in p53 mRNA (*p* < 0.001) compared with gentamicin alone according to the *post hoc* Tukey test in the ANOVA model. Expression of p53 targets Wig-1, p21, and Mdm2 also showed a significant increase (*p* < 0.05) in mRNA levels following combination treatment compared with single treatment with gentamicin. Statistical analysis was done using the Wilcoxon matched pairs test since the distributions differed significantly from the normal one. Nutlin-3a itself did not induce any substantial increase in mRNA levels of p53 nor p53 target genes. Since G418 shows stronger readthrough effect than gentamicin and since bortezomib has higher general toxicity than nutlin-3a, we focused on the combination of G418 and nutlin-3a for the following studies. According to Western blotting (Figure 3C), the effect of the combined treatment with nutlin-3a and G418 on p21 expression is dependent on p53, since H1299 cells carrying empty vector did not show any increase in p21.

In addition, we tested if G418, nutlin-3a, and G418 in combination with nutlin-3a would affect the expression of p53 and p53 target genes in wild-type p53-carrying HCT116 cells. G418 itself did not cause any substantial induction of p53 or p53 target genes in the HCT116 wtp53^+/+^ cells, except at the highest concentration (400 µM) where a modest induction of p53 and p21 was observed (Figure S1A in Supplementary Material). This could be due to cellular toxicity since we observed increased cell death at this concentration. As expected, nutlin-3a alone induced p53 protein but not mRNA in the HCT116 cells, and induced p53 target genes at both protein and mRNA levels (Figures S1B,C in Supplementary Material). G418 did not further enhance induction of p53 protein by nutlin-3a, although we observed increased levels of p21 and Fas mRNA upon combination treatment with nutlin-3a and G418. The differences between nutlin-3a-treated and control samples were statistically significant (*p* < 0.001) for Mdm2, p21, and Fas mRNA according to the *post hoc* Tukey multiple comparison in the ANOVA model (Figure S1C in Supplementary Material).

### Induction of Full-Length p53 Elicits a Biological Response in H1299 Cells

**Figure 4:**
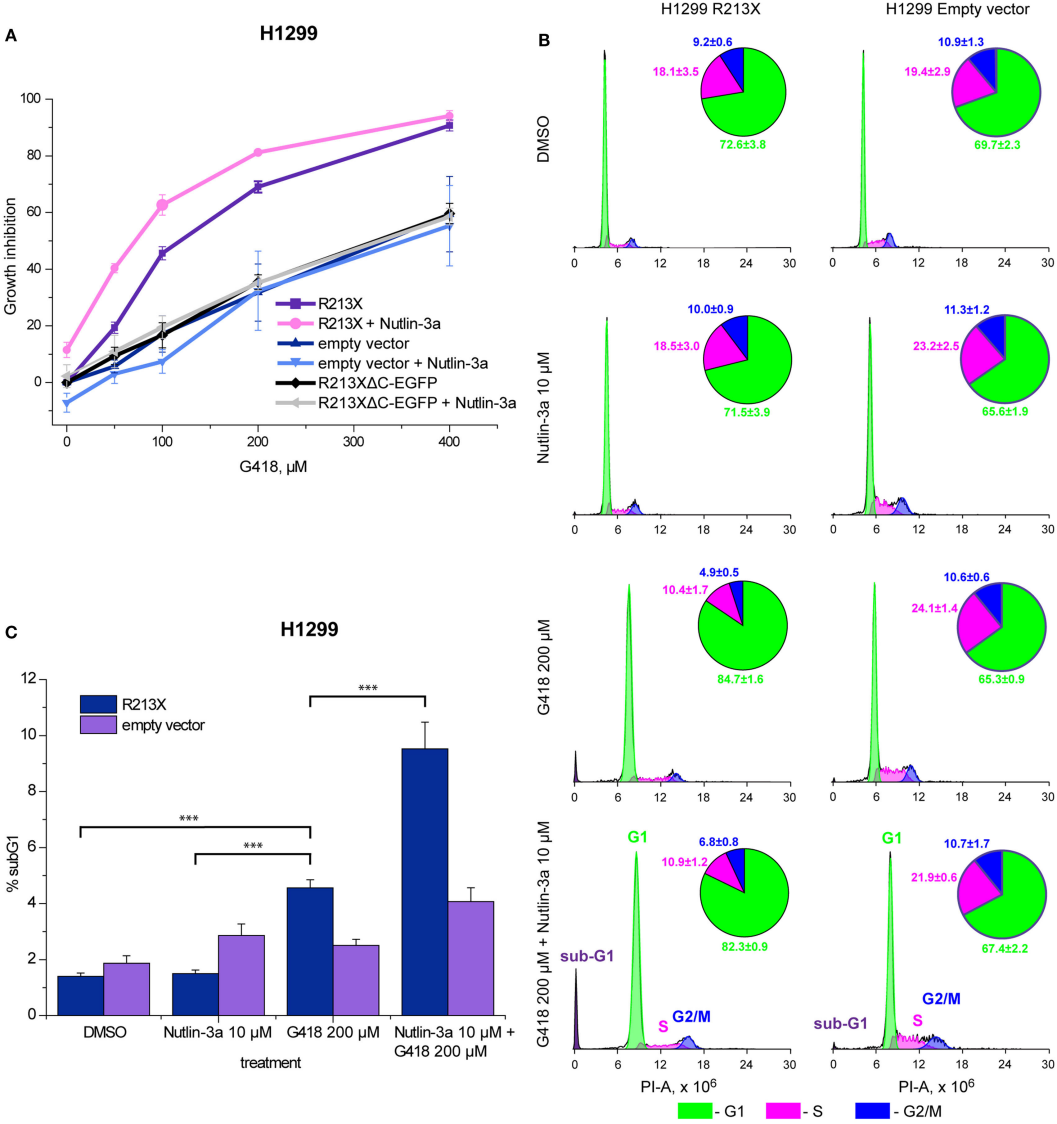
Combination treatment with nutlin-3a potentiates G418-induced p53-dependent growth suppression and cell death in H1299 cells. **(A)** Inhibition of growth of R213X-transfected H1299 cells and empty vector control cells as assessed by the WST-1 assay. Three independent experiments were performed in triplicates. Mean of each experiment was used for statistical analysis. Significant difference (*p* < 0.05) in growth inhibition between H1299 R213X and H1299 empty vector cells was shown for all concentrations using the non-parametric Kruskal–Wallis test. **(B)** Analysis of cell cycle arrest by FACS-PI. Treatment with G418 resulted in a significant increase (*p* < 0.05) in the G1 fraction of cells in cells carrying R213X mutant p53 but not in empty vector control cells. The unequal N HSD *post hoc* test in the ANOVA model was used for statistical analysis. Four independent experiments were performed with seven observations for each treatment and cell type. **(C)** Increased sub-G1 population upon treatment with G418 and nutlin-3a alone or in combination as shown by FACS-PI. Four independent experiments were carried out with seven observations for each treatment and cell type. The unequal N HSD *post hoc* test in the ANOVA model was used for statistical analysis (*** = *p* < 0.001).

We further studied the ability of full-length p53 induced by aminoglycosides to inhibit cell growth and trigger cell death in H1299 cells carrying the R213X p53 construct. First, we assessed the effect on cell growth using the WST-1 proliferation assay. Induction of full-length p53 by G418 resulted in a marked inhibition of cell growth (Figure 4A). Cells carrying the R213XΔ*C*-EGFP fusion construct or empty vector were less sensitive to G418, confirming that the observed effect is dependent on full-length p53. We observed statistically significant differences (*p* < 0.05) in G418-induced growth inhibition between H1299 cells carrying the R213X p53 construct and H1299 cells carrying empty vector according to the Kruskal–Wallis test. We also examined the cell cycle distribution after treatment with G418. As shown in Figure 4B, G418 induced G1 cell cycle arrest in the R213X-carrying H1299 cells but had no major effect in the H1299 empty vector control cells, indicating that the induction of G1 arrest by G418 is p53-dependent. The difference in G1 population between G418-treated and control R213X-carrying H1299 cells was statistically significant (*p* < 0.05), while the difference between G418-treated and control H1299 empty vector cells was not significant according to the Unequal N HSD *post hoc* test in the ANOVA model test.

### Combination Treatment Potentiates Growth Suppression and Cell Death in H1299 Cells

We finally tested the effect of the combination of aminoglycosides and nutlin-3a on cell growth and survival in H1299 cells carrying R213X mutant p53. Analysis of cell growth by the WST-1 assay demonstrated a significant increase in growth suppression upon treatment with G418 in combination with nutlin-3a in H1299-R213X cells, when compared with treatment with G418 alone. The difference between combination treatment and G418 alone at 50 µM was statistically significant in H1299-R213X cells (*p* < 0.05) but not in H1299 empty vector cells according to the *post hoc* Tukey analysis in the ANOVA model. This indicates significant synergy (Figure 4A). Importantly, nutlin-3a did not induce any further growth suppression in the H1299 R213XΔ*C*-EGFP and empty vector control cells, confirming that this synergistic growth-inhibitory effect is p53-dependent.

Moreover, we analyzed the effect of the combined treatment with G418 and nutlin-3a on cell cycle distribution by FACS-PI. As shown in Figure 4B, nutlin-3a did not enhance G1 arrest induced by G418. However, nutlin-3a was potent in enhancing the induction of a sub-G1 cell population in combination with G418 in H1299-R213X cells when compared with control or nutlin-3a treated cells. The difference reached statistical significance (*p* < 0.001) according to the Unequal N HSD *post hoc* test in the ANOVA model (Figure 4C). This synergistic effect on sub-G1 population induction is p53-dependent, since we did not observe any synergistic increase in H1299 empty vector control cells. Thus, we observed a statistically significant increase in sub-G1 population for both G418 and G418 in combination with nutlin-3a in H1299-R213X cells (*p* < 0.05, unequal N HSD *post hoc* test in ANOVA model).

## Discussion

Aminoglycoside antibiotics have previously been shown to induce translational readthrough of nonsense mutant *TP53* and expression of full-length p53 protein (29). One important question is whether full-length p53 induced by G418 or gentamicin is functional. In agreement with (29), we found that induction of full-length p53 was associated with upregulation of p53 targets such as *CDKN1A* (*P21*), *BAX, FAS, MDM2*, and *WIG-1*, arguing that the protein has retained the specific DNA binding and transactivation function of wild-type p53. However, we cannot conclude based on our results that all full-length p53 molecules induced by translational readthrough have a wild-type p53 sequence. It is conceivable that translational readthrough produces a mixture of p53 proteins with different amino acids inserted at codon 213, such as Leu (UUA), Ser (UCA), Cys (UGU and UGC), Trp (UGG), Arg (AGA), and Gly (GGA), all of which have codons that only show one mismatch with the PTC at position 213 (UGA). A recent study indicated that Arg, Trp, and Cys are the most frequently inserted amino acids upon G418-induced readthrough of UGA stop codons in human cells (38). The fact that there are no known missense mutations at *TP53* codon 213 in human tumors (7, 8, 39) suggests that substitutions at codon 213 do not have any major effect on p53 function. Nonetheless, it seems likely that there is some degree of functional heterogeneity among the p53 proteins generated upon readthrough of the R213X nonsense mutation. It is interesting to note that G418 and gentamicin induced somewhat different patterns of p53 target gene expression, which could possibly be related to differences in the spectrum of inserted amino acids at codon 213.

We observed that G418 and gentamicin induced levels of both full-length and truncated p53 protein. One possible reason for the increased levels of truncated p53 is stabilization of p53 mRNA through inhibition of NMD upon induction of translational readthrough. The level of nonsense mutant p53 mRNA is normally low since any mRNA with PTCs would be degraded by NMD (11, 28). Induction of translational readthrough of a PTC leads to inhibition of NMD and stabilization of the mRNA, due to removal of NMD-triggering exon-junction complexes during translation (27, 35). This is consistent with our finding that p53 mRNA levels were strongly increased by aminoglycosides (see also below). Our previous data demonstrated that the p53 target Wig-1 can stabilize p53 mRNA in a positive feedback loop (40). Thus, we hypothesized that induction of Wig-1 by full-length p53 could contribute to increased p53 mRNA levels upon readthrough induction by aminoglycosides. However, our analysis of p53 mRNA levels upon Wig-1 siRNA knockdown or Wig-1 overexpression in HDQ-P1 cells or H1299 cells did not indicate any major effect of Wig-1 on p53 mRNA in these cells (Palomar-Siles et al., unpublished).

With the aim of identifying possible combination treatment strategies that would allow sub-toxic doses of aminoglycosides and thus less severe side effects, we tested combination of aminoglycosides with agents that prevent p53 degradation. We tested both the proteasome inhibitor bortezomib and the Mdm2 inhibitors nutlin-3a and MI-773. Bortezomib has a general stabilizing effect on many cellular proteins while Mdm2 inhibitors specifically stabilize p53 by inhibiting Mdm2-mediated p53 ubiquitination and subsequent proteasomal degradation. Indeed, we observed that bortezomib increased levels of full-length p53 upon aminoglycoside treatment. The combination with nutlin-3a and MI-773 was even more effective in boosting p53 levels and the p53 response. Mechanistically, this effect is well in line with the observed induction of Mdm2 mRNA in HDQ-P1 cells treated with aminoglycosides (Figure 2A). In other words, expression of full-length and functionally active p53 leads to induction of Mdm2, which will stimulate p53 degradation and thereby downregulate p53 levels. We also observed that the combination of aminoglycosides with nutlin-3a stabilized p53 mRNA, while nutlin-3a alone had very little or no effect, consistent with the lack of full-length p53 upon single treatment with nutlin-3a.

Earlier studies have shown more potent aminoglycoside-induced readthrough of the *TP53* R213X mutation when compared with other *TP53* nonsense mutations (29, 41). We tested readthrough-inducing effect of G418 in several human tumor cell lines carrying different endogenous *TP53* nonsense mutations. Only HDQ-P1 cells showed strong induction of readthrough and expression of full-length p53, whereas CACO-2 colorectal carcinoma cells carrying E204X nonsense mutant *TP53* showed relatively modest readthrough (Zhang et al., unpublished), in agreement with previous results.

An important question is whether full-length p53 induced by aminoglycosides, alone or in combination with Mdm2 inhibitors, is biologically active. We found that treatment with G418 alone resulted in significant p53-dependent growth inhibition, and G1 cell cycle arrest and cell death. The combination of G418 with nutlin-3a resulted in an even stronger growth inhibition and induction of cell death. However, it is interesting to note that nutlin-3a did not enhance G418-induced G1 cell cycle arrest to any major extent (Figure 4B), even though it caused a substantial increase in p21 mRNA and protein expression upon G418 treatment. One possible explanation is that the levels of p21 induced by full-length p53 upon G418 treatment are sufficient to induce potent G1 arrest in the H1299 cells, and therefore a further increase in p21 levels would not lead to any further increase in G1 arrest. It is also possible that the G1 cell population has started to undergo apoptosis, which would result in a lower proportion of G1-arrested cells as the G1 cells move to the sub-G1 fraction.

## Conclusion

Treatment with aminoglycosides G418 and gentamicin as single agents leads to significant translational readthrough of R213X nonsense mutant *TP53* and expression of full-length p53 that is functional as assessed by upregulation of p53 target genes and induction of growth suppression and cell death. Importantly, combination treatment with aminoglycosides and proteasome inhibitor bortezomib or p53-Mdm2 inhibitors nutlin-3a or MI-773 allowed a synergistic increase in full-length p53 levels and significant potentiation of the biological response. These results suggest that combination treatment with low-dose aminoglycosides and p53-Mdm2 inhibitors should be explored further as a possible strategy for treatment of tumors that carry *TP53* nonsense mutations.

## Author Contributions

KW and VB were responsible for the experimental design. MZ, AH, MP-S, and SÖ performed experiments. All authors took part in analysis and interpretation of data. All authors also contributed to writing of the manuscript and approved the final version.

## Conflict of Interest Statement

VB and KW are co-founders and shareholders of Aprea Therapeutics AB, a company that develops p53-based cancer therapy including APR-246. KW is a member of its Clinical Advisory Board. Research in the KW lab has received financial support from Aprea Therapeutics AB. KW has received salary from Aprea Therapeutics AB.

## Supplementary Material

Figure S1 Effect of G418 alone and G418 in combination with nutlin-3a on expression of p53 and p21 in HCT116 wtp53^+/+^ cells. **(A)** Western blot analysis showing that G418 alone at concentrations up to 200 µM does not induce wild-type p53 or p21 protein levels. A modest induction of p53 and p21 is detected at 400 µM G418. The blots were cut at 25 kDa, the upper part was first probed with p53 antibody DO-1 and then stripped and blotted with antibodies against GAPDH. The lower part was blotted with p21 antibody. **(B)** Western blot analysis showing that G418 does not induce a further increase in p53 and p21 protein levels in nutlin-3a-treated cells. The blots were cut at 25 kDa, the upper part was first probed with p53 antibody DO-1 and then stripped and blotted with GAPDH antibody. The lower part was blotted with p21 antibody. **(C)** Real-time PCR analysis showing that G418 alone or G418 in combination with nutlin-3a has no effect on wild-type p53 mRNA levels in HCT116 wtp53^+/+^ cells. Nutlin-3a significantly induces mRNA levels of p53 targets Mdm2, p21, and Fas. Combination treatment with G418 and nutlin-3a causes a further increase in p21 and Fas mRNA levels. β-actin and c1/c2 were used as negative controls. Three independent experiments were performed in duplicates. The parametric Tukey post hoc test in the ANOVA model (*** = *p* < 0.001) was used for statistical analysis.

## Abbreviations

APC, adenomatous polyposis coli; ATM, ataxia telangiectasia mutated; NMD, nonsense-mediated decay; PTC, premature termination codon; TP53, tumor suppressor p53; CFTR, cystic fibrosis transmembrane conductance regulator; DMD, Duchenne muscular dystrophy.
