# Supplementary figures and images for: Synergistic Rescue of Nonsense Mutant Tumor Suppressor p53 by Combination Treatment with Aminoglycosides and Mdm2 Inhibitors
Source: Front Oncol. 2018 Jan 4;7:323. doi: 10.3389/fonc.2017.00323 (PMC5758538; doi:10.3389/fonc.2017.00323)

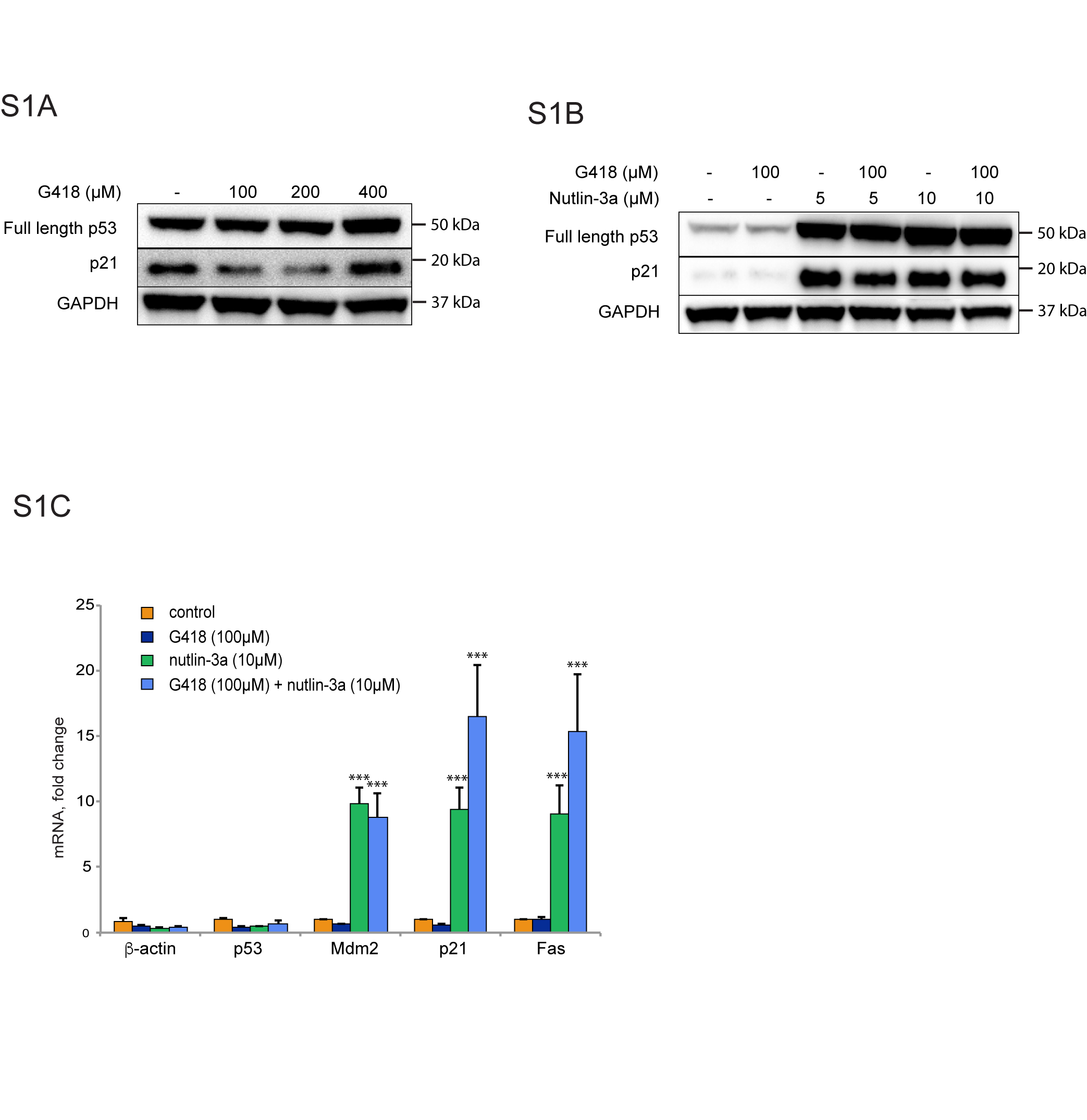

Supplement: Figure S1 — Effect of G418 alone and G418 in combination with nutlin-3a on expression of p53 and p21 in HCT116 wtp53+/+ cells. (A) Western blot analysis showing that G418 alone at concentrations up to 200 µM does not induce wild-type p53 or p21 protein levels. A modest induction of p53 and p21 is detected at 400 µM G418. The blots were cut at 25 kDa, the upper part was first probed with p53 antibody DO-1 and then stripped and blotted with antibodies against GAPDH. The lower part was blotted with p21 antibody. (B) Western blot analysis showing that G418 does not induce a further increase in p53 and p21 protein levels in nutlin-3a-treated cells. The blots were cut at 25 kDa, the upper part was first probed with p53 antibody DO-1 and then stripped and blotted with GAPDH antibody. The lower part was blotted with p21 antibody. (C) Real-time PCR analysis showing that G418 alone or G418 in combination with nutlin-3a has no effect on wild-type p53 mRNA levels in HCT116 wtp53+/+ cells. Nutlin-3a significantly induces mRNA levels of p53 targets Mdm2, p21, and Fas. Combination treatment with G418 and nutlin-3a causes a further increase in p21 and Fas mRNA levels. β-actin and c1/c2 were used as negative controls. Three independent experiments were performed in duplicates. The parametric Tukey post hoc test in the ANOVA model (*** = p < 0.001) was used for statistical analysis. [file Image_1.jpeg]
